# Characterizing adult rehabilitation programs for solid organ transplant candidates and recipients across Canada

**DOI:** 10.3389/fresc.2025.1674381

**Published:** 2025-11-27

**Authors:** Sahar Sohrabipour, Nicholas Bourgeois, Sunita Mathur, Tania Janaudis-Ferreira, Sherrie Logan, Lisa Wickerson, Dmitry Rozenberg

**Affiliations:** 1 Temerty Faculty of Medicine, University of Toronto, Toronto, ON, Canada; 2 Division of Respirology, Toronto Lung Transplant Program, Ajmera Transplant Centre, Toronto General Hospital, UHN, Toronto, ON, Canada; 3 Lung Transplant Program, Centre Hospitalier de l’Université de Montréal, Montréal, QC, Canada; 4 School of Rehabilitation, University of Montreal, Montréal, QC, Canada; 5 School of Rehabilitation Therapy, Queen’s University, Kingston, ON, Canada; 6 Research Institute of McGill University Health Centre, Montreal, QC, Canada; 7 Canadian Donation and Transplantation Research Program, Edmonton, AB, Canada; 8 Department of Physical Therapy, University of Toronto, Toronto, ON, Canada; 9 West Park Health Care Centre, University Health Network, Toronto, ON, Canada

**Keywords:** solid organ transplantation, rehabilitation, telerehabilitation, exercise training, physical activity

## Abstract

**Introduction:**

Rehabilitation is integral for solid organ transplant (SOT) candidates and recipients, and aims to build physical capacity for surgery, facilitate post-operative recovery, and mitigate long-term complications. Prior to the COVID-19 era, in-person programs were the primary delivery model in Canadian SOT rehabilitation programs, but there are several knowledge gaps with the current delivery models. The aims of this study were to: 1) assess the characteristics and current practices of SOT rehabilitation programs in Canada, and 2) identify key facilitators and barriers to providing rehabilitation for the SOT population.

**Methods:**

An electronic survey was administered to 17 adult Canadian SOT rehabilitation programs utilizing REDCap in April 2024. The survey examined types of exercise training and supervision practices, clinical outcome measures, delivery models, safety considerations, facilitators, and barriers. Survey measures were summarized using descriptive statistics.

**Results:**

The response rate was 59% (10/17). Post COVID-19, there has been a shift in program delivery, with majority (60%) of SOT rehabilitation programs now using a hybrid approach comprised of both in-person and virtual components. There is heterogeneity among programs with respect to clinical assessments, safety measures, and virtual rehabilitation platforms. The most common barriers were limitations in funding and healthcare personnel.

**Conclusion:**

This study provides a better understanding of the current landscape and variability of SOT rehabilitation programs. Most programs have transitioned to hybrid models post-COVID-19, which may facilitate greater access. Future research can leverage findings from this survey to optimize SOT rehabilitation programs and improve clinical outcomes.

## 1 Introduction

Solid organ transplant (SOT) is a lifesaving procedure globally for many individuals with end-stage organ failure. The main goals of SOT are to improve quality of life, physical function, and independence in activities of daily living, which is supported by pre- and post-transplant rehabilitation (1, 2). Rehabilitation aims to improve individuals' functional abilities, quality of life, and clinical outcomes through various modalities, including exercise training, education, mental health and nutritional support (3). The rehabilitation benefits have been observed throughout the transplant journey: pre-transplant, early post-transplant (i.e., up to 6 months after transplant), and late post-transplant (i.e., long-term self-management). This is important given the high prevalence of limitations in exercise capacity and limb muscle dysfunction following SOT (4).

Exercise-based rehabilitation has been shown to improve functional outcomes in SOT transplant candidates and recipients, including improvements in exercise capacity, muscle strength, and quality of life (3, 5–8). There is an increasing need for rehabilitation given the complexities of transplantation, including increased wait times for SOT candidates and increased co-morbidities, physical frailty and older age of transplant candidates (9, 10). Physical deconditioning from advanced organ disease may contribute to limb muscle weakness, weight loss, and cachexia, which are risk factors for increased morbidity (11). Furthermore, prehabilitation has been shown to improve physical function, which is associated with reduced hospital length of stay (3, 12–14), and fewer re-hospitalizations post-transplant (15, 16). Exercise training also reduces morbidity and mortality in transplant recipients (17). Despite the benefits of rehabilitation programs for SOT candidates and recipients, there are no standardized clinical practice guidelines (4, 6).

In 2010, a survey was conducted across SOT transplant centers in Canada to determine the availability, characteristics, and barriers to providing in-person outpatient rehabilitation for individuals pre- and post-transplant (6). The key barriers highlighted were lack of funding and health care personnel (6). Further, there were no reported rehabilitation programs for kidney transplant patients, and only one rehabilitation program was identified for liver transplant patients. All SOT clinical rehabilitation programs were delivered in-person as per the 2010 survey (6). However, due to restrictions with in-person exercise during the COVID-19 pandemic, most rehabilitation programs transitioned to a virtual delivery model (i.e., telerehabilitation) or a hybrid delivery model (i.e., comprised of both in-person and telerehabilitation) (18), leading to a variety of practices across Canada (19–21). Moreover, there is variation in rehabilitation delivery with respect to the technology used for virtual care and remote patient monitoring, type of supervision, equipment needs, and online programs used to implement telerehabilitation (22). A better understanding of the existing programs available, including access, delivery, barriers, and facilitators to rehabilitation, will help facilitate pre- and post-transplant management.

The study was conducted to survey outpatient SOT rehabilitation programs across Canada to understand the evolution of SOT rehabilitation over the last 15 years. The objectives of this study were: 1) To assess the characteristics, practices, and delivery of rehabilitation programs for SOT patients in Canada pre- and post-transplant, and 2) To identify key facilitators and barriers to providing rehabilitation for SOT candidates and recipients.

## 2 Methods

### 2.1 Study design

A cross-sectional survey was administered electronically through Research Electronic Data Capture (REDCap), a secure online survey tool, in April 2024. An invitation letter, consent form, and survey were developed in both English and French (Canada's official languages), and sent to the 17 adult SOT rehabilitation programs listed on a national website, Canadian Network for Rehabilitation and Exercise for Solid Organ Transplant Optimal Recovery (CAN-RESTORE) (23). CAN-RESTORE is continuously revised to reflect a list of active and available SOT rehabilitation programs in Canada. These 17 identified programs have been verified by CAN-RESTORE in offering pre- and post-transplant rehabilitation. This method of recruitment differed from the 2010 survey as the CAN-RESTORE website was not available in 2010 (6). This national network focuses on communication, advocacy, resource sharing, and developing capacity within the healthcare system for the delivery of transplant exercise programs (23).

The invitation letter, which contained a link to the questionnaire and consent form, was sent to the email address of the contact person listed on CAN-RESTORE for each of the rehabilitation programs. If an email address was not available, SOT rehabilitation programs were contacted by telephone to inquire about a contact email address. To increase survey response rates, a modified Dillman approach was utilized, which consists of a respondent-friendly survey, multiple contact attempts (up to two emails and up to two phone call follow-ups), and personalized correspondence (i.e., addressing the health care provider by name in emails) (24).

The survey was comprised of 40 questions (Supplementary Methods), and was piloted by seven individuals including clinicians, researchers, physiotherapists, and partners with lived experience who provided feedback to ensure its clarity and comprehensiveness. To increase response completeness and improve participant comfort, all questions had both “not applicable” and “prefer not to answer” response options. Ethics approval was obtained from the University Health Network (Research Ethics Board #23-5671.0).

### 2.2 Inclusion and exclusion criteria

Participants were invited to complete the survey if they were a health care provider with sufficient knowledge about the SOT rehabilitation program at their institution and if the program met the following inclusion criteria: 1) adult SOT program that provides rehabilitation (main component of structured exercise training) for single- or multi-organ transplant patients (lung, heart, kidney, liver, pancreas) pre- and/or post-transplant, and 2) SOT rehabilitation program is offered by a transplant centre, rehabilitation centre, and/or hospital in Canada. The exclusion criteria were as follows: 1) rehabilitation program provides services only for non-solid organ transplant recipients (e.g., stem cells, bone marrow), and 2) SOT program had no contact information (email and/or a phone number) of an English- or French-speaking health care provider listed on the CAN-RESTORE website.

### 2.3 Statistical analysis

Descriptive statistics were undertaken for all key survey measures stemming from the questionnaires to help characterize the SOT rehabilitation programs. Statistical analyses were performed using GraphPad Prism (Version 8.4.3). The chi-square test for trend was used to compare differences in the mode of rehabilitation delivery, and a two-tailed *p* < 0.05 was considered to be significant.

## 3 Results

### 3.1 Rehabilitation program characteristics and practices

Twenty adult SOT rehabilitation programs were identified across Canada from the CAN-RESTORE website, and the survey was sent to 17 programs (3 programs did not have a valid email address or phone number). Ten programs responded to our survey (59% response rate; 10/17). The programs reported providing rehabilitation for the following transplant patient populations: heart (*n* = 3), lung (*n* = 6), liver (*n* = 5), kidney/kidney-pancreas (*n* = 8), and small bowel/multi-visceral (*n* = 1). Compared to 2010, there have been a qualitative increase in the number of rehabilitation programs available for kidney and liver transplant (Supplementary Table S1). Rehabilitation was offered during the following transplant periods: pre-transplant (90%; 9/10), early post-transplant (i.e., < 6 months post-transplant) (80%; 8/10), and late post-transplant (60%; 6/10). Participation in the rehabilitation program was a mandatory part of the transplant requirements for 50% (5/10) of programs in the pre-transplant period, and for 40% (4/10) of programs in the post-transplant period. Additional details on the demographics of SOT rehabilitation programs, including the duration of rehabilitation provided and the number of individuals seen per week, are provided in Supplementary Table S2.

Reported characteristics of current SOT rehabilitation practices within the past six months are shown in Table 1. Details on the exercise prescription components (frequency, intensity, type, and time) are summarized in Supplementary Table S3, and information on wearable devices and the collection of health data (heart rate, daily steps, and oxygen saturation) are reported in Supplementary Table S4.

**Table 1:** Reported SOT rehabilitation practices.

Characteristics	SOT rehabilitation programs (*n* = 10)
In-person delivery model	
Group sessions	5 (50%)
One-on-one sessions	4 (40%)
Not applicable/prefer not to answer	1 (10%)
Telerehabilitation delivery model	
Online synchronous group sessions	3 (30%)
Online synchronous individual sessions	1 (10%)
Online asynchronous (i.e., not supervised in real time)	2 (20%)
Not applicable/prefer not to answer	4 (40%)
Platform(s) used to deliver telerehabilitation*	
Video-based application (e.g., Zoom, Microsoft Teams)	5 (50%)
Phone calls	4 (40%)
Website-based (e.g., websites with instructional exercise videos)	2 (20%)
App-based (e.g., application individuals download on their device)	2 (20%)
Patient requirements for their own monitoring and/or video technology	
Yes, required	5 (50%)
No, rehabilitation program provides all required technology	0 (0%)
Not applicable/prefer not to answer	5 (50%)
Patient requirement for own exercise equipment	
Yes, required	1 (10%)
No, program provides all required exercise equipment	1 (10%)
No, exercise equipment can be adapted based on items at home (e.g., using water bottles or cans for weights)	5 (50%)
Not applicable/prefer not to answer	3 (30%)
Safety measure(s) for telerehabilitation delivery*	
Health care provider having patient's contact information	6 (60%)
Ensuring individuals are comfortable using technology	6 (60%)
Performing an initial in-person assessment by health care provider to ensure adequate space and safe environment to perform exercises	5 (50%)
Guidelines for red flags and action plan on when to call a health care provider if issues arise	5 (50%)
Ensuring individuals have webcams on at all times during exercises (if providing a synchronous exercise session)	4 (40%)
Ensuring individuals have another person present with them when exercising	3 (30%)
Requirement for baseline in-person functional assessment	2 (20%)
Requiring that individuals have certain exercise equipment or medical grade monitors	2 (20%)

Results are shown as proportions, *n* (%), with all listed *n* values being out of 10.

* Percentages did not add up to 100% for questions with response options that were not mutually exclusive (i.e., multiple response options were selected by one program).

**Figure 1:**
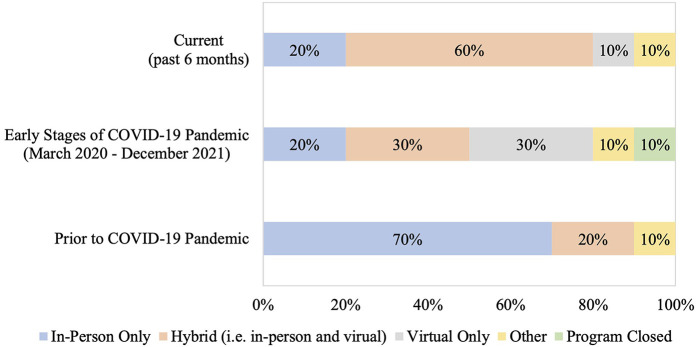
Method of rehabilitation delivery prior to the COVID-19 pandemic, during the early stages of the pandemic, and currently. Current refers to the 6-month period prior to when the survey was administered, early stages of the COVID-19 pandemic is defined from March 2020 to December 2021, and prior to the COVID-19 pandemic refers to the period before March 2020. Other category represents: exercise booklet and education provided by a physiotherapist. The chi-square test for trend was used to compare differences in rehabilitation delivery prior to the COVID-19 pandemic and currently (*p* = 0.15). Comparisons were not done for the early stages of the COVID-19 pandemic due to different categorical options (i.e., “program closed”).

There was variability in the mode of delivery among SOT rehabilitation programs prior to the COVID-19 pandemic, during the early stages of the pandemic, and presently (i.e., within the past 6 months) (Figure 1). Prior to the pandemic, majority of SOT rehabilitation programs were offered in-person only (70%; 7/10), whereas the most common mode of rehabilitation delivery currently (i.e., past 6 months) was a hybrid model of delivery (60%; 6/10). In 2010, rehabilitation programs were offered exclusively in-person, whereas only 20% (2/10) programs are currently offered in-person only (Supplementary Table S1).

### 3.2 Functional assessments and services provided to patients

SOT rehabilitation programs reported completing various functional assessments with individuals during their initial visit as well as after completion of the program, including exercise capacity, muscle strength, frailty, body composition, quality of life, and patient-reported outcomes (Supplementary Figure 1). These initial assessments were completed in-person by 70% (7/10) of rehabilitation programs, and in a hybrid format (i.e., both in-person and virtual) by 30% (3/10) of programs. The most common functional assessment performed was exercise capacity, while patient-reported outcome measures evaluated with questionnaires was completed by only one program.

After completing the exercise training program, 60% (6/10) of SOT rehabilitation programs reported that they had a maintenance program for individuals, such as community programs or follow-up visits with the rehabilitation team, which provided opportunities for counselling and post-transplant care education. Details on the duration of these programs and the number of individuals seen per week are provided in Supplementary Table S2. Additionally, programs reported offering the following resources and guidance for long-term self-management: exercise program prescription (90%; 9/10), health care team follow-up (50%; 5/10), and an educational booklet (40%; 4/10).

In addition to exercise training, rehabilitation programs reported offering the following services to patients: nutritional support (100%; 10/10), patient education (100%; 10/10), mental health support (90%; 9/10), and referrals to other programs, services, or health care providers (60%; 6/10). These services were offered during the pre-transplant period (in 90% of programs; 9/10), early post-transplant (80%; 8/10), and late post-transplant (60%; 6/10). Additional details about these services, such as examples provided by the SOT rehabilitation programs, are provided in Supplementary Table S5.

### 3.3 Rehabilitation program barriers and facilitators

**Figure 2:**
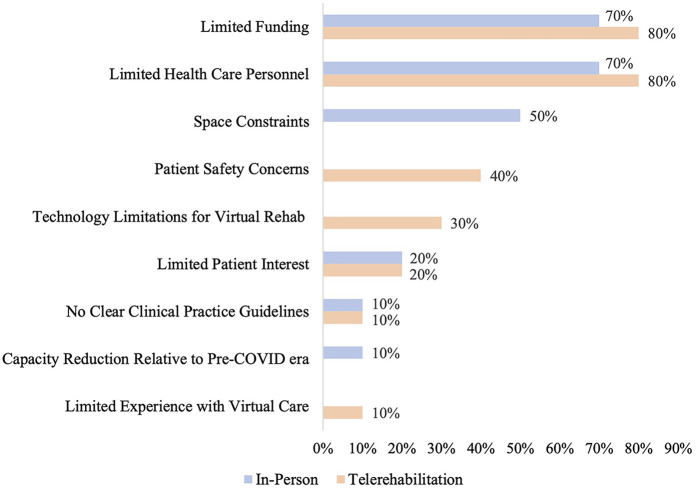
Reported barriers to providing in-person and/or telerehabilitation. Barriers that were not applicable to either in-person or telerehabilitation are represented by empty bars on the graph. Patient privacy concerns were also a response option for barriers to providing telerehabilitation, but were not selected by any program.

Programs were asked about the current barriers they experience in providing in-person and telerehabilitation. The three most commonly reported barriers to providing in-person rehabilitation were funding constraints (70%; 7/10), limited health care personnel (70%; 7/10), and lack of space to accommodate high patient volumes (50%; 5/10). For telerehabilitation, the most commonly cited barriers were funding constraints (80%; 8/10), limited health care personnel (80%; 8/10), and patient safety concerns (40%; 4/10) (Figure 2).

**Figure 3:**
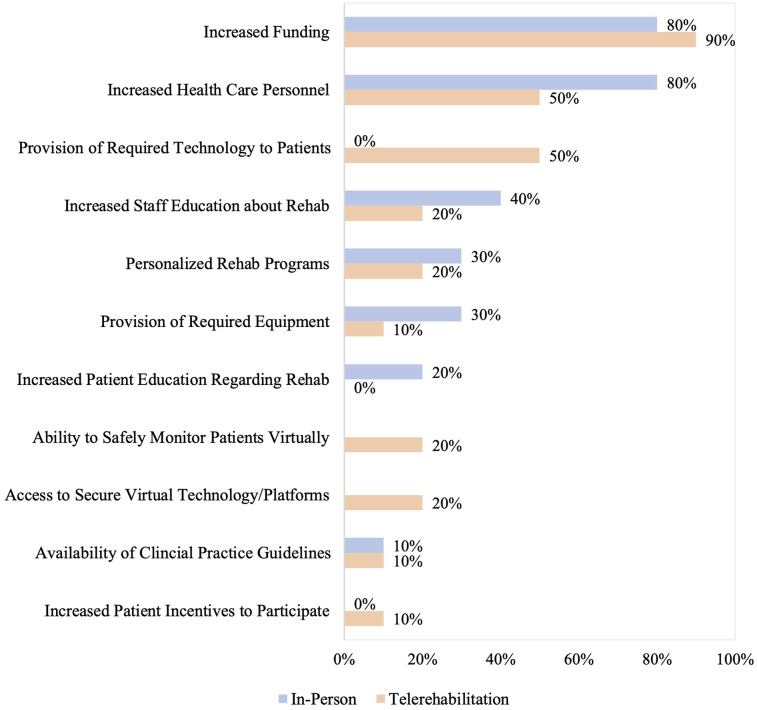
Reported facilitators to providing in-person and/or telerehabilitation. Facilitators that were not applicable to either in-person or telerehabilitation are left as empty bars on the graph. Facilitators that were not selected by any programs, despite being response options, are reported as 0%.

Respondents were also asked to select the top three facilitators for providing in-person and telerehabilitation. The three most common facilitators to providing in-person rehabilitation were increased funding (80%; 8/10), health care personnel (80%; 8/10), and staff education regarding rehabilitation (40%; 4/10). For telerehabilitation, increased funding (90%; 9/10), health care personnel (50%; 5/10), and provision of required technology for patients (50%; 5/10) were the most common facilitators, as shown in Figure 3.

## 4 Discussion

The present study provides insight into the current landscape of SOT rehabilitation in Canada, and highlights the changes in practices over the past 15 years. Most programs have shifted towards hybrid models of SOT rehabilitation post-COVID-19, with increased availability of liver and kidney programs compared to 2010 (6). However, significant heterogeneity exists across SOT rehabilitation programs with respect to program delivery, online platforms, patient assessments, and individual requirements to participate, leading to a variety of practices across Canada. Despite these differences, key barriers of limited funding and health care personnel were similar across programs, and to the 2010 survey (6).

Following the COVID-19 pandemic, the majority of programs transitioned to delivering SOT rehabilitation using a hybrid model, whereas most programs had been offered in-person previously (6). This shift can have many benefits and help increase access to rehabilitation, especially for individuals who do not have reliable transportation to healthcare centers (25–27). A recent study found that pre- and post-lung transplant patients with cystic fibrosis preferred hybrid delivery models for rehabilitation, as it offers increased flexibility to exercise at home, while in-person sessions can be used to initially prescribe and progress exercises in a safe manner (28). Moreover, Heindl et al. reported that a hybrid cardiac rehabilitation program increased patient participation rates, improved exercise capacity, quality of life, and reduced costs associated with program delivery, compared to in-person programs (18, 29). Similarly, Esayed et al. concluded that hybrid models improve follow-up care in kidney transplant recipients by enhancing patient-centered care through improved access and flexibility (29).

Telerehabilitation, which can be delivered through either a hybrid or fully virtual model, may pose several challenges. Individuals without the required equipment, technological access, or lack of familiarity with online platforms may have difficulties accessing telerehabilitation programs (30, 31). Further, half of the programs surveyed in this study reported that patients were required to have their own video technology to participate in telerehabilitation, which may be a financial barrier for some individuals. In a systematic review by Velez and colleagues, the costs associated with video technology and internet accessibility were identified as barriers, which may pose restrictions for individuals with lower incomes requiring rehabilitation services (32). A study of 166 lung transplant recipients participating in hybrid rehabilitation reported that 52% of patients had low participation rates, with higher numbers of in-person sessions completed compared to telerehabilitation (33). While additional research is needed in this area, some studies suggest that individuals with frailty may be another population that could derive greater benefit from in-person rehabilitation programs. In-person rehabilitation helps with review of exercise techniques and training progression, which are important for optimizing health-related outcomes (34, 35).

This study highlights the heterogeneity that exists across SOT rehabilitation programs in Canada. Specifically, there are significant variations in the online tools used to deliver rehabilitation (e.g., video-based or website-based), in-person delivery models (e.g., group vs. one-on-one sessions), and virtual delivery modalities (e.g., synchronous vs. asynchronous sessions). Determining the optimal rehabilitation delivery models and practices remains an active area of research. A recent 2-day Canadian meeting attended by SOT clinicians, researchers, patients, and family partners evaluated current practices and identified future research priorities (36). The key research priorities highlighted were the optimal delivery and timing of rehabilitation programs, the use of digital tools and wearable devices to monitor patient safety and progress, and optimizing clinical assessments to track outcomes (36).

The survey responses highlighted that a variety of assessments are being used across rehabilitation programs to evaluate clinical outcomes (e.g., exercise capacity tests, measures of muscle strength, and health-related quality of life). Similarly, a mix of different wearable devices (e.g., smart watches, pedometers, and pulse oximeters) are also being utilized. While identifying optimal practices can allow for standardization of SOT rehabilitation programs, some studies support the creation of flexible programs that can be personalized to each individual's unique needs (37, 38). For example, Damery et al. describes a hybrid cardiac rehabilitation application (Active^+^me REMOTE) that has the flexibility to be tailored to support individual circumstances with good uptake (39). Individuals were able to choose between in-person or telerehabilitation (39). This flexibility can empower patients by improving access to rehabilitation based on individual preferences. Further, smartphone applications (e.g., Heal-Me and EL-FIT) have also been developed to support transplant candidates and recipients by promoting exercise training and rehabilitation in participants' location of choice (i.e., home or in-person) (40, 41).

The current survey identified that SOT rehabilitation programs apply several safety measures when delivering virtual exercise training. Prior to rehab initiation, programs familiarize individuals with technology, perform initial in-person home assessments to ensure a safe exercise environment, and have guidelines and action plans in place in the event of a health emergency. These strategies are consistent with proposed facilitators during an SOT telerehabilitation consensus meeting (36). Other important safety considerations for telerehabilitation, as noted in the literature, include re-evaluating patients who experience a change in their health status prior to resuming their program, requiring a caregiver to be present during training sessions, and educating individuals on recognizing early warning signs for exercise cessation (e.g., chest pain or presyncope) (36, 42, 43). Further, technological advancements such as wearable devices or sensors capable of alerting health care providers and/or emergency personnel of medical instability (e.g., unstable vital signs) can be utilized as a safety measure with telerehabilitation (44).

Improving rehabilitation programs and associated clinical outcomes requires an initial understanding of the barriers and facilitators that programs currently experience, which our survey sought to accomplish. The most common barriers highlighted for both in-person and telerehabilitation were limitations in funding and health care personnel, which were consistent with the 2010 survey (6). Increasing funding and health care personnel could help with the development of SOT rehabilitation programs, especially in underserviced areas. Previous studies have also suggested that increased funding can be used for hiring and training staff, and expanding rehabilitation services such as mental health and nutritional support (36, 45, 46). Another common barrier reported with in-person program was limited space to accommodate high patient volumes, which could be mitigated by greater uptake of telerehabilitation programs. This is in contrast to the 2010 survey, which observed a low volume of patients as a common barrier to rehabilitation implementation (6). Additional benefits of telerehabilitation include more flexibility in timing of exercise sessions (especially for asynchronous models), lower infection risks, and reduced costs, distance, and time associated with travelling (36, 47, 48). Similarly, a systematic review highlighted that telerehabilitation is more cost-effective compared to in-person programs, with most of the costs of virtual programs being attributed to technology and equipment needs (49). In fact, provision of required technology to patients and increased funding were the most frequently selected facilitators of telerehabilitation among the programs in our survey.

There are several limitations in this study that should be highlighted. First, we surveyed rehabilitation programs across Canada, and so this resulted in a small sample size, and may have limited generalizability. However, Canada has a publicly funded health care system whereby individuals can freely access medical services, including rehabilitation (50). As such, the study results may be quite applicable to other countries with publicly funded health care systems. Further, we sent the survey to all verified rehabilitation programs identified on the CAN-RESTORE website. Second, we may have omitted some programs if they were not listed on the CAN-RESTORE website. However, we aimed to mitigate omissions by asking rehabilitation programs that we contacted in the initial invitation letter if they routinely refer patients to other SOT rehabilitation programs so that we can also survey those programs. No additional programs were identified in this manner. Further, the directory of programs on the website is routinely updated to maintain a current list of available SOT rehabilitation programs in Canada. While individuals may attend other programs and clinics offering rehabilitation, there should be some direction from transplant programs, which is reflected on the CAN-RESTORE website. Third, lack of response may have introduced bias into our results; however, we had diverse representation with at least one response from each province that we surveyed across the country. Finally, our study only surveyed health care providers, and so future research studies can focus on understanding patient-reported perspectives and outcomes to complement health care provider data. These future directions would be invaluable for optimizing patient-centered SOT rehabilitation programs.

## 5 Conclusion

In conclusion, this national survey provides insight into current SOT rehabilitation programs across Canada, and their evolution over the past 15 years. The most common reported barriers for both in-person and telerehabilitation programs were constraints in funding and health care personnel, which have not changed since the 2010 survey (6). However, there have been major changes to how programs are delivered after the COVID-19 pandemic with transition from in-person to hybrid delivery models, with significant heterogeneity in practices across programs. The heterogeneity identified in this study is crucial to informing future directions and development of standardized delivery models. Taken together, this study serves as an important step to help identify optimal program delivery models that expand opportunities for future clinical and research collaborations, with the goal of improving patient-centered outcomes, program adherence, and satisfaction with SOT rehabilitation.

## Data Availability

The original contributions presented in the study are included in the article/Supplementary Material, further inquiries can be directed to the corresponding author.
