# Supplementary material for: Characterizing adult rehabilitation programs for solid organ transplant candidates and recipients across Canada
Source: Front Rehabil Sci. 2025 Nov 27;6:1674381. doi: 10.3389/fresc.2025.1674381 (PMC12695816; doi:10.3389/fresc.2025.1674381)
Supplement: Supplementary file 1 [file Datasheet1.pdf]

Supplementary Material

Table of Content

Supplementary Methods – Survey (Pages 2–10)
Supplementary Table S1. Key qualitative comparisons between 2010 and current SOT rehabilitation programs. (Page 11)
Supplementary Table S2. Demographics of SOT rehabilitation programs. (Page 12)
Supplementary Table S3. SOT rehabilitation program exercise training frequency, intensity, type, and time. (Page 13)
Supplementary Table S4. Wearable devices and collection of health data. (Page 14)
Supplementary Table S5. Examples of services provided to patients by SOT rehabilitation programs. (Page 15)
Supplementary Figure 1. Functional assessment performed in the SOT rehabilitation programs. (Page 16)

<u>Supplementary Methods – Survey</u>

Section 1: Demographics

1. Which province or territory is the solid organ transplantation (SOT) rehabilitation program located in?
    a. British Columbia
    b. Alberta
    c. Saskatchewan
    d. Manitoba
    e. Ontario
    f. Quebec
    g. New Brunswick
    h. Nova Scotia
    i. Prince Edward Island
    j. Newfoundland and Labrador
    k. Yukon
    l. Northwest Territories
    m. Nunavut
    n. Prefer not to answer

2. The program provides rehabilitation for which of the following solid organ transplant(s)? (*please select all that apply)*
    - ☐ Heart
    - ☐ Kidneys
    - ☐ Liver
    - ☐ Lungs
    - ☐ Pancreas
    - ☐ Small Bowel
    - ☐ Other, *please specify:* _____
    - ☐ Prefer not to answer

3. The program provides rehabilitation for SOT patients during which stage of transplant? *(please select all that apply)*
    - ☐ Pre-transplant
    - ☐ Early post-transplant (i.e. < 6 months post-transplant)
    - ☐ Late post-transplant (i.e. > 6 months post-transplant)
    - ☐ Prefer not to answer

4. What is the total duration of the rehabilitation program for SOT patients? *(please select all that apply)*
    - ☐ Pre-transplant rehabilitation duration *(please specify):* _____
    - ☐ Early post-transplant rehabilitation duration *(please specify):* _____
    - ☐ Late post-transplant rehabilitation duration *(please specify):* _____
    - ☐ Not applicable
    - ☐ Prefer not to answer

5. **Pre-transplant**, how many patients (new and/or follow-up) attend the rehabilitation program per week, on average? *Please include the total number of patients for both the in-person and virtual program.*
    a. 0 – 9
    b. 10 – 19
    c. 20 – 30
    d. > 30
    e. Unsure
    f. Not applicable
    g. Prefer not to answer

6. **Pre-transplant**, is participation in the rehabilitation program a mandatory part of the transplant requirements?
    a. Yes
    b. No
    c. Prefer not to answer

7. **Post-transplant**, how many patients (new and/or follow-up) attend the rehabilitation program per week, on average? *Please include the total number of patients for both the in-person and virtual program.*
    a. 0 – 9
    b. 10 – 19
    c. 20 – 30
    d. > 30
    e. Unsure
    f. Not applicable
    g. Prefer not to answer

8. **Post-transplant**, is participation in the rehabilitation program a mandatory part of the transplant requirements?
    a. Yes
    b. No
    c. Prefer not to answer

Section 2: Program Delivery, Technology, and Safety

9. As part of the exercise-based rehabilitation program, which of the following additional services are offered to patients? *(please select all that apply)*
    ☐ Nutritional support, *please provide additional details:* _____
    ☐ Mental health support, *please provide additional details:* _____
    ☐ Patient education, *please provide additional details:* _____
    ☐ Referral to other programs, services, or health care providers, *please provide additional details:* _____
    ☐ Other, *please specify:* _____
    ☐ Not applicable
    ☐ Prefer not to answer

10. When are the services listed in question 9 offered? *(please select all that apply)*
    ☐ Pre-transplant

☐ Early post-transplant (i.e. < 6 months post-transplant)
☐ Late post-transplant (i.e. > 6 months post-transplant)
☐ Not applicable
☐ Prefer not to answer

11. Which of the following best characterizes the rehabilitation program's mode of delivery for exercise training **prior to the COVID-19 pandemic (prior to March 2020)**?
    a. In-person only
    b. Virtual only
    c. Hybrid (in-person and virtual components), *please specify the percentage of in-person vs. virtual:* _____
    d. Other, *please specify:* _____
    e. Unsure
    f. Prefer not to answer

12. Which of the following best characterizes the changes that occurred to the rehabilitation program during the **early stages (March 2020 to December 2021)** of the COVID-19 pandemic?
    a. The program closed
    b. The program continued in-person with modifications, *please provide additional details about the modifications (e.g. reduced capacity, no group sessions):* _____
    c. The program transitioned to virtual only
    d. The program transitioned to hybrid (in-person and virtual components)
    e. No change
    f. Other, *please specify:* _____
    g. Unsure
    h. Prefer not to answer

13. Which of following best characterizes the rehabilitation program's **current** (past 6 months) mode of delivery for exercise training?
    a. In-person only
    b. Virtual only
    c. Hybrid (in-person and virtual components), *please specify the percentage of in-person vs. virtual:* _____
    d. Other, *please specify:* _____
    e. Prefer not to answer

14. Which of the following **initial** patient functional assessments are completed? (*please select all that apply)*
    ☐ Exercise Capacity Tests (e.g. Six-Minute Walk Test, Step Test), *please specify:* _____
    ☐ Body Composition Measurements (e.g. Body Mass Index), *please specify:* _____
    ☐ Frailty Measurements (e.g. Fried frailty phenotype), *please specify:* _____
    ☐ Measures of Muscle Strength (e.g. Sit to Stand Test, One-Repetition Maximum Test), *please specify:* _____
    ☐ Health-Related Quality of Life (e.g. Short Form-36 Survey), *please specify :* _____
    ☐ Patient-Reported Outcome Measures (e.g. patient questionnaires), *please specify:* _____

- ☐ Other, *please specify*: _____
- ☐ Not applicable
- ☐ Prefer not to answer

15. How are **initial** patient functional assessments completed?
    a. In-person only
    b. Virtual only
    c. Hybrid (in-person and virtual), *please specify how decisions are made about assessing patients in-person vs. virtually*: _____
    d. Other, *please specify:* _____
    e. Prefer not to answer

16. How are patient outcomes measured **throughout and/or after** the rehabilitation program? (*please select all that apply)*
    - ☐ Exercise Capacity Tests (e.g. Six-Minute Walk Test, Step Test), *please specify:* _____
    - ☐ Body Composition Measurements (e.g. Body Mass Index), *please specify:* _____
    - ☐ Frailty Measurements (e.g. Fried frailty phenotype), *please specify:* _____
    - ☐ Measures of Muscle Strength (e.g. Sit to Stand Test, One-Repetition Maximum Test), *please specify:* _____
    - ☐ Muscle Endurance, *please specify*: _____
    - ☐ Health-Related Quality of Life (e.g. Short Form-36 Survey), *please specify:* _____
    - ☐ Patient-Reported Outcome Measures (e.g. patient questionnaires), *please specify:* _____
    - ☐ Other, *please specify*: _____
    - ☐ Not applicable
    - ☐ Prefer not to answer

17. What is the structure of the rehabilitation program's **in-person** exercise training sessions?
    a. One-on-one sessions with patients
    b. Group sessions, *please specify how many people per session:* _____
    c. Other, *please specify*: _____
    d. Not applicable
    e. Prefer not to answer

18. If the program has a virtual component, how is exercise training delivered?
    a. Online synchronous (i.e. supervised by health care provider in real-time) group session
    b. Online synchronous (i.e. supervised by health care provider in real-time) individual session
    c. Online asynchronous (i.e. unsupervised)
    d. Other, *please specify:* _____
    e. Not applicable
    f. Prefer not to answer

19. If the program has a virtual component, which of the following platform(s) are used to deliver rehabilitation? *(please select all that apply)*
    - ☐ Video-based application (e.g. Zoom, Microsoft Teams)

- ☐ Website-based platform (i.e. websites with instructional exercise videos or exercise information), *please specify the name of the platform:* _____
- ☐ App-based platform (i.e. application patients download on their device), *please specify the name of the platform:* _____
- ☐ Phone calls
- ☐ Other, *please specify:* _____
- ☐ Not applicable
- ☐ Prefer not to answer

20. If the program has a virtual component, what patient safety measures/guidelines are in place? *(please select all that apply)*
    - ☐ Performing an initial in-person assessment by health care professional to ensure adequate space and safe environment to perform exercises
    - ☐ Requirement for baseline in-person functional assessment
    - ☐ Guidelines for red flags and action plan on when to call a health care provider if issues arise
    - ☐ Health care provider having patient's contact information
    - ☐ Ensuring patients are comfortable using technology
    - ☐ Ensuring patients have webcams on at all times during exercises (if providing a synchronous exercise session)
    - ☐ Ensuring patients have another person present with them when exercising
    - ☐ Requiring that patients have certain exercise equipment or medical grade monitors
    - ☐ Other, *please specify:* _____
    - ☐ Not applicable
    - ☐ Prefer not to answer

21. As part of the rehabilitation program's exercise training, do patients use wearable devices (e.g. smart watches, pulse oximeters)? *(please select all that apply)*
    - ☐ Smart watch (e.g. Fitbit, Apple, Samsung)
    - ☐ Pedometers
    - ☐ Pulse oximeter
    - ☐ Heart rate monitor
    - ☐ Other, *please specify:* _____
    - ☐ Not applicable
    - ☐ Prefer not to answer

22. If patients use wearable devices as part of the rehabilitation program, do patients record health data **outside exercise sessions** (e.g. heart rate, oxygen saturation)? *(please select all that apply)*
    - ☐ Heart rate
    - ☐ Oxygen saturation
    - ☐ Blood pressure
    - ☐ Daily steps
    - ☐ Other (e.g. sedentary time), *please specify:* _____
    - ☐ Not applicable
    - ☐ Prefer not to answer

23. If patients record health data as part of the rehabilitation program, is this data shared with their healthcare providers?
    a. Yes, *please specify how patients share health data (e.g. verbally during synchronous exercise, manually by recording, sending data on patient portal):* _____
    b. No
    c. Not applicable
    d. Prefer not to answer

24. Do patients have to have their own monitoring and/or video technology to participate in the rehabilitation program?
    a. Yes, *please specify which (e.g. Internet/WiFi, laptop/computer, webcam):* _____
    b. No, program provides all required technology, *please specify which:* _____
    c. Not applicable
    d. Prefer not to answer

25. Do patients have to have their own exercise equipment to participate in the rehabilitation program? (*please select all that apply)*
    a. Yes, patients have to purchase exercise equipment, *please specify which:* _____
    b. No, program provides all required exercise equipment, *please specify which:* ___
    c. No, exercises/program can be adapted based on items that patients have at home (e.g. using water bottles or cans for weights), *please provide examples:* ___
    d. Not applicable
    e. Prefer not to answer

26. After completing the exercise training program, is there a maintenance program (i.e. follow-up/counselling/education provided to patients)?
    a. Yes, *please provide additional details:* _____
    b. No
    c. Prefer not to answer

27. After completing the exercise training program, what resources or guidance are patients provided with for long-term self-management? *(please select all that apply)*
    - ☐ Exercise program prescription
    - ☐ Education manual
    - ☐ Ongoing counselling
    - ☐ Follow-up with health care team
    - ☐ Other resources, *please specify:* _____
    - ☐ Not applicable
    - ☐ Prefer not to answer

Section 3: Frequency, Intensity, Type, Time (FITT) Principle for Rehabilitation Program

Questions 28 – 31 Pertain to Pre-Transplant Rehabilitation

28. **Pre-transplant**, what type of exercise training does the rehabilitation program provide patients? *(please select all that apply)*
    - ☐ Aerobic/endurance training
    - ☐ Strength/resistance training
    - ☐ Flexibility/stretching exercises
    - ☐ Inspiratory muscle training

- ☐ Other, *please specify:* _____
- ☐ Not applicable
- ☐ Prefer not to answer

29. **Pre-transplant**, how often are patients prescribed exercise training? *Please answer based off the <u>most</u> number of days per week that patients are engaging in all types of exercise (aerobic, strength, and/or flexibility training)*
    a. 1-2 days per week
    b. 3-4 days per week
    c. 5 or more days per week
    d. Other (e.g. if patients are prescribed a biweekly training schedule), *please specify*: _____
    e. Not applicable
    f. Prefer not to answer

30. **Pre-transplant**, what is the duration of each exercise session (please combine total time spent doing aerobic, resistance, and/or flexibility training per session)?
    a. 0-45 minutes
    b. 46-90 minutes
    c. > 90 minutes
    d. Other, *please specify*: _____
    e. Not applicable
    f. Prefer not to answer

31. **Pre-transplant**, what is the intensity prescribed for exercise training for aerobic and/or resistance training (e.g. % of heart rate maximum, one-repetition maximum)?
    a. Low-intensity, *please specify measurement:*_____
    b. Moderate-intensity, *please specify measurement*: _____
    c. High-intensity, *please specify measurement*: _____
    d. Other, *please specify*: _____
    e. Not applicable
    f. Prefer not to answer

Questions 32 – 35 Pertain to Post-Transplant Rehabilitation

32. **Post-transplant**, what type of exercise training does the rehabilitation program provide patients? *(please select all that apply)*
    - ☐ Aerobic/endurance training
    - ☐ Strength/resistance training
    - ☐ Flexibility/stretching exercises
    - ☐ Inspiratory muscle training
    - ☐ Other, *please specify:* _____
    - ☐ Not applicable
    - ☐ Prefer not to answer

33. **Post-transplant**, how often are patients prescribed exercise training? *Please answer based off the <u>most</u> number of days per week that patients are engaging in all types of exercise (aerobic, strength, and/or flexibility training)*

a. 1-2 days per week
b. 3-4 days per week
c. 5 or more days per week
d. Other (e.g. if patients are prescribed a biweekly training schedule), *please specify*: _____
e. Not applicable
f. Prefer not to answer

34. **Post-transplant**, what is the duration of each exercise session (please combine total time spent doing aerobic, resistance, and/or flexibility training per session)?
    a. 0-45 minutes
    b. 46-90 minutes
    c. > 90 minutes
    d. Other, *please specify*: _____
    e. Not applicable
    f. Prefer not to answer

35. **Post-transplant**, what is the intensity prescribed for exercise training for aerobic and/or resistance training (e.g. % of heart rate maximum, one-repetition maximum)?
    a. Low-intensity, *please specify measurement*: _____
    b. Moderate-intensity, *please specify measurement*: _____
    c. High-intensity, *please specify measurement*: _____
    d. Other, *please specify*: _____
    e. Not applicable
    f. Prefer not to answer

Section 4: Barriers and Facilitators of a Rehabilitation Program
Questions 36 – 37 Pertain to In-Person Rehabilitation

36. What are some barriers your program **currently** experiences with providing **in-person** rehabilitation? (*please select all that apply)*
    - ☐ Limited funding
    - ☐ Lack of space to accommodate high patient volumes
    - ☐ Lack of patients in a centralized region/lack of patient interest
    - ☐ Limited health care personnel
    - ☐ No clear clinical practice guidelines for exercise
    - ☐ Capacity reduction relative to pre-COVID era
    - ☐ Other, *please specify:* _____
    - ☐ Not applicable
    - ☐ Prefer not to answer

37. Please select what the **top 3** facilitators would be to providing **in-person** rehabilitation from the following list:
    - ☐ Increased funding
    - ☐ Increased health care personnel
    - ☐ Provision of required equipment (e.g. dumbbells) to patients
    - ☐ Provision of required technology (e.g. wearable devices) to patients
    - ☐ Availability of clinical practice guidelines
    - ☐ Increased staff education regarding rehabilitation

- ☐ Increased patient education regarding rehabilitation
- ☐ Increased patient incentives to participate
- ☐ Tailoring rehabilitation programs to meet individual patient goals and needs
- ☐ Other, *please specify:* _____
- ☐ Not applicable
- ☐ Prefer not to answer

Questions 38 – 39 Pertain to Virtual Rehabilitation

38. What are some barriers your program **currently** experiences with providing **virtual** rehabilitation? (*please select all that apply)*
    - ☐ Lack of funding
    - ☐ Limited health care personnel
    - ☐ Lack of patient interest
    - ☐ Lack of technology to deliver virtual rehabilitation
    - ☐ Patient safety concerns
    - ☐ Patient privacy concerns
    - ☐ Lack of experience with virtual care
    - ☐ Lack of clinical practice guidelines
    - ☐ Other, *please specify:* _____
    - ☐ Not applicable
    - ☐ Prefer not to answer

39. Please select what the **top 3** facilitators would be to providing **virtual** rehabilitation from the following list:
    - ☐ Increased funding
    - ☐ Increased health care personnel
    - ☐ Provision of required equipment (e.g. dumbbells) to patients
    - ☐ Provision of required technology (e.g. wearable devices) to patients
    - ☐ Availability of clinical practice guidelines
    - ☐ Increased staff education regarding rehabilitation
    - ☐ Increased patient education regarding rehabilitation
    - ☐ Increased patient incentives to participate
    - ☐ Tailoring rehabilitation programs to meet individual patient goals and needs
    - ☐ Ability to safely monitor patients virtually
    - ☐ Access to secure virtual technology/platforms to deliver rehabilitation
    - ☐ Other, *please specify:* _____
    - ☐ Not applicable
    - ☐ Prefer not to answer

Other Feedback

40. Would you like to provide us with any other feedback/comments/suggestions regarding any of the previous survey questions?
    a. Yes - Text box
    b. No

Supplementary Results

Table S1. Key qualitative comparisons between 2010 and current SOT rehabilitation programs.

	**2010* (n = 12)**	**Current (n = 10)**
**Availability of Rehabilitation Programs**		
Kidney	0/12 (0%)	8/10 (80%)
Liver	1/12 (8%)	5/10 (50%)
Lung	5/12 (42%)	6/10 (60%)
Heart	6/12 (50%)	3/10 (30%)
**Mode of Program Delivery**		
In-Person only	12/12 (100%)	2/10 (20%)
Hybrid (i.e. in-person and virtual)	0/12 (0%)	6/10 (60%)
Virtual only	0/12 (0%)	1/10 (10%)
Other (i.e. exercise booklet and education provided by a physiotherapist)	0/12 (0%)	1/10 (10%)

Results are shown as proportions (percentage), n (%).
*Reference:
Trojetto T, Elliott RJ, Rashid S, et al. Availability, characteristics, and barriers of rehabilitation programs in organ transplant populations across Canada. *Clin Transplant*. 2011;25(6):E571-E578. doi:10.1111/j.1399-0012.2011.01501.x

Table S2. Demographics of SOT rehabilitation programs.

**Demographics**	**SOT Rehabilitation Programs (n =10)**
**Pre-transplant: duration of rehabilitation provided**	
Throughout the entire transplant listing period	4 (40%)
6 months or less	3 (30%)
Not applicable/prefer not to answer	3 (30%)
**Post-transplant: duration of rehabilitation provided**	
Up to 3 months post-transplant	4 (40%)
Up to 6 months post-transplant	2 (20%)
Other	
From time of transplant to discharge home	1 (10%)
Up to 3 months post-transplant and annual follow-up	1 (10%)
Not applicable/prefer not to answer	2 (20%)
**Pre-transplant: number of patients who attended rehabilitation program**	
0-9 patients per week	7 (70%)
10-19 patients per week	1 (10%)
More than 30 patients per week	1 (10%)
Not applicable/prefer not to answer	1 (10%)
**Post-transplant: number of patients who attended rehabilitation program**	
0-9 patients per week	5 (50%)
10-19 patients per week	0 (0%)
20-30 patients per week	2 (20%)
Unsure	2 (20%)
Not applicable/prefer not to answer	1 (10%)

Results are shown as proportions, n (%), with all listed n values being out of 10.

Table S3. SOT rehabilitation program exercise training frequency, intensity, type, and time.

Exercise Training Session Frequency, Intensity, Type, Time (FITT) Principles	SOT Rehabilitation Programs (n = 10)
**Pre-transplant frequency**	
3-4 days per week	3 (30%)
5 or more days per week	4 (40%)
Personalized schedule	2 (20%)
Not applicable/prefer not to answer	1 (10%)
**Pre-transplant intensity**	
Moderate intensity	5 (50%)
Personalized	2 (20%)
Not applicable/prefer not to answer	3 (30%)
**Pre-transplant type(s) of exercise training***	
Aerobic/endurance	9 (90%)
Strength/resistance	9 (90%)
Flexibility/stretching	7 (70%)
Inspiratory muscle training	2 (20%)
**Pre-transplant time spent doing all types of exercise training**	
0-45 minutes	2 (20%)
46-90 minutes	4 (40%)
Personalized duration	3 (30%)
Not applicable/prefer not to answer	1 (10%)
**Post-transplant frequency**	
3-4 days per week	4 (40%)
5 or more days per week	4 (40%)
Personalized schedule	1 (10%)
Not applicable/prefer not to answer	1 (10%)
**Post-transplant intensity**	
Moderate intensity	6 (60%)
Personalized	2 (20%)
Not applicable/prefer not to answer	2 (20%)
**Post-transplant type(s) of exercise training***	
Aerobic/endurance	9 (90%)
Strength/resistance	9 (90%)
Flexibility/stretching	8 (80%)
Inspiratory muscle training	3 (30%)
**Post-transplant time spent doing all types of exercise training**	
0-45 minutes	2 (20%)
46-90 minutes	5 (50%)
More than 90 minutes	1 (10%)
Personalized duration	1 (10%)
Not applicable/prefer not to answer	1 (10%)

Results are shown as proportions, n (%), with all listed n values being out of 10.
*Percentages did not add up to 100% for questions with response options that were not mutually exclusive (i.e. multiple response options were selected by one program).

Table S4. Wearable devices and collection of health data.

**Use of wearable device(s) during rehabilitation program**	
Yes	5*
Pulse oximeter	5 (100%)
Smart watch	3 (60%)
Heart rate monitor	2 (40%)
Pedometer	1 (20%)
Health data from device(s) is shared with health care providers	4 (80%)
**Collection of health data collected outside of exercise training sessions**	
Yes	5*
Heart rate	4 (80%)
Daily steps	4 (80%)
Oxygen saturation	2 (40%)

Results are shown as proportions, n (%), with all listed n values being out of 5.
*Represents the number of rehabilitation programs that responded "yes" for each question. The specific responses provided by programs that responded "yes" are listed above (e.g. pulse oximeter, smart watch).

Table S5. Examples of additional services provided to patients by SOT rehabilitation programs.

**Services/Supports Provided by SOT Rehabilitation Program**	**Examples Reported**
Nutritional Support	• Follow-up/referral to a registered dietician and/or nutritionist<br>• Consultations/handouts provided regarding healthy eating
Patient Education	• Education provided by rehabilitation team on various topics, such as smoking cessation, weight loss, pre-operative health optimization, and self-care<br>• Educational handouts and video clips were also provided to patients
Mental Health Support	• Referral to a psychiatrist, psychologist, or social worker<br>• Stress management course provided<br>• Mindfulness, relaxation, and behavioral support
Referral to Other Programs, Services, or Health Care Providers	• Referral to other specialties as required, such as geriatrics, physiatry, palliative care, complex pain management, and addictions specialists

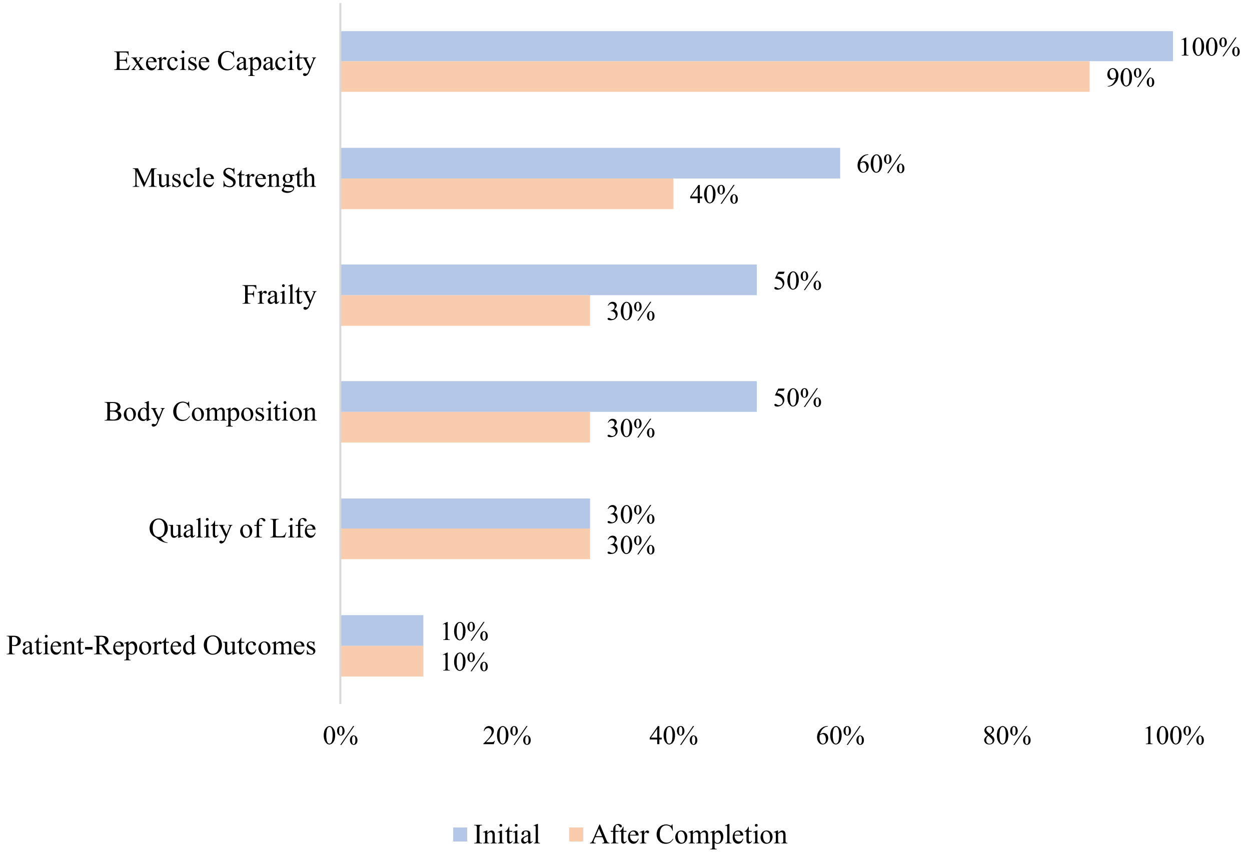

Supplementary Figure 1. Functional assessment performed in the SOT rehabilitation programs.
